# Mediterranean Diet as a Tool to Combat Inflammation and Chronic Diseases. An Overview

**DOI:** 10.3390/biomedicines8070201

**Published:** 2020-07-08

**Authors:** Christina Tsigalou, Theocharis Konstantinidis, Afroditi Paraschaki, Elisavet Stavropoulou, Chrissoula Voidarou, Eugenia Bezirtzoglou

**Affiliations:** 1 Laboratory of Microbiology, Medical School, Democritus University of Thrace, 68100 Dragana, Alexandroupolis, Greece; tkonsta@med.duth.gr (T.K.); aparashaki@gmail.com (A.P.); 2 Centre Hospitalier Universitaire Vaudois (CHUV), Rue du Bugnon, Vaud, CH-1011 Lausanne, Switzerland; elisabeth.stavropoulou@gmail.com; 3 Public Health Laboratory, 47100 Arta Prefecture, Arta, Greece; xvoidarou@yahoo.gr; 4 Laboratory of Hygiene and Environmental Protection, Medical School, Democritus University of Thrace, 68100 Dragana, Alexandroupolis, Greece; empezirt@med.duth.gr

**Keywords:** diet, Mediterranean diet, health, inflammation, diet patterns, nutrients, diet habit, chronic diseases

## Abstract

Since ancient times, the quality of nourishment is a milestone for the maintenance of health and as it is stated ‘prevention is better than cure’, amongst the so-called ‘healthy’ diets Mediterranean diet (MD) claims the lion’s share. It stands in good stead because of a variety of valuable macro- and micronutrients. So, adherence to a MD is associated with the reduction of inflammation and non-communicable (NCD) OR chronic diseases. Numerous studies try to scrutinize the role of MD components as regards reducing inflammation, lowering rate, and mortality for disorders and illnesses, and preventing NCD. MD regime of the inhabitants of the Mediterranean basin includes a variety of ethnic nutritional habits and regulates an array of effects and epigenetic changes that affect human wellbeing. The research is still ongoing and endeavors to elucidate every aspect of this issue. This review focuses on the impact of MD on inflammation highlights positive results regarding NCD and indicates the need for more high-quality experiments and trials in order to overcome any discrepancies.

## 1. Introduction

The positive impact of food on health was postulated by the ancient Hippocrates, father of modern medicine with his famous quote: “Let food be thy medicine and medicine be thy food” [1]. In the 21st century, scientists have focused on the effect of nutritional habits in diseases. Nowadays, it is well documented that food plays a noteworthy role in the pathogenesis of chronic diseases namely cardiovascular diseases (CVD), metabolic syndrome, diabetes mellitus type II, and cancer [2,3,4,5], as it correlates with others with the lipid pattern, the blood pressure, and the endothelial function. The scientists examine the effect of nutritional habits on disease emergence and progression in both individual nutrient intake and dietary patterns models. Worldwide, two dietary patterns are usually compared—the Mediterranean diet (MD) and the Western diet (WD) [3,6]. The Mediterranean diet reflects the food culture of most Mediterranean countries based on olive oil consumption, seasonal fresh vegetables, cereals, and plants in balance with low consumption of meat [7]. The Western diet, on the contrary, is dominated by high-fat dairy products processed and red meat [8]. However, discordance in the different MD patterns and consumed food doses had been recognized. Without any doubt, those discrepancies could confine and restrict our knowledge on the health benefit mechanisms of the MD [9]. Due to the above, the medical community along with nutritionists and dieticians take a keen interest in MD and its traits [7].

In the present review, we aim to summarize accruing evidence regarding the Mediterranean diet and its beneficial effects upon health by focusing on its anti-inflammatory mechanisms.

## 2. Mediterranean Diet and Inflammation

Mediterranean diet is a healthy eating custom that emphasizes fruits, vegetables, olive oil consumption, and limited meat eating. Previous studies have shown that daily adherence to MD has a positive effect on cardiovascular diseases, diabetes, arthritis, cancer, and obstructive sleep apnea [10,11], and is also associated with a significant reduction of total mortality [12]. The advantages of MD adoption are already known for almost 50 years since the diminished cardiovascular disease risk was firstly observed in Mediterranean people correlated to their nutritional habits [13,14]. Moreover, in the past 20 years, all the new knowledge from implementation of cutting-edge technologies in microbiology offered a bulk of information about the immense significance of the gut microbiome. But till today although we understand that gut microbiota composition is of paramount importance for human health and we also realize the connection to the MD, the actual pathways by which that happens are not fully elucidated [15].

Considering as a fact that inflammation could play a significant and sometimes malicious role undermining the host’s health it is obvious from 2 new studies that people adopting the MD had actually less inflammation. Both elegant studies from Mesliel et al., and Ghosh TS et al., capitalizing on gut microbiome’s metabolomics and metagenomics, supported the importance of food quality and not quantity and also that even in the elderly there are obvious benefits in metabolism and inflammation [16,17]. The efficacy of MD on inflammation was also previously described namely Bonaccio M et al., in their research, which included 14,586 healthy subjects, and they reported that White Blood Count and platelets counts were both inversely related to MD adherence (*p* = 0.008, and *p* < 0.0001 respectively) [18]. Moreover, MD down-regulates cellular and humoral immunological pathways related to disease activity and progression. Additionally, Mena M. et al., in their study presented that MD inhibits the recruitment and adhesion of PBMCs to the endothelium through downregulation of CD 40 expression on the PBMCs, exhibiting a molecular anti-inflammatory action (*p* = 0.0044) [19].

## 3. Role of Mediterranean Diet Components on Inflammatory Response

### 3.1. Trace Elements and Minerals Contribution

As it was previously shown MD adherence directly correlated with trace elements dietary intake (Fe, Cu, Fe, Zn, Mo, Na, K, Ca, Mg) and also that it is more abundant in factors which can affect the absorption of minerals such as Zn [20]. It is of interest to note that low serum micronutrient levels were observed in patients with Major Depressive Disorder and although the etiopathogenic role of trace elements in different disease states should be furtherly studied, it is possible that MD could benefit them. Moreover, it seems that there is an association between the serum trace element levels and developmental dysplasia of the hip [21,22,23].

### 3.2. Salt Intake

High salt (sodium chloride, NaCl) content in the Western diet is implicated via the hyperosmotic stress in inflammatory response. Osmotic stress can induce the release of proinflammatory cytokines from human mononuclear cells in culture. Mice on a high-salt diet developed a more severe course of EAE (experimental autoimmune encephalomyelitis) that was associated with a pronounced TH17 response in vivo, in an SGK1- (serum/glucocorticoid-regulated kinase 1 SGK1) and IL-23R-dependent manner [24,25]. Over-salting diet disrupts the activation of macrophages (M2) which weakens the tissue inflammation processes and promotes wound healing [26]. Furthermore, over-salting seems to block the Treg function by inducing IFNγ production in the cells. In this vein, it is showed that environmental signals under over-salting diet increase proinflammatory responses through innate and adaptive regulatory mechanisms [26]. In contrast, higher adherence to the MD which means low salt intake could be the attributing parameter for the inverse association with hypertension [27].

### 3.3. The Olive Oil Treasure

Olive oil is the ultimate pillar of the Mediterranean Diet. Oleocanthal is a minor constituent of olive oil with strong anti-inflammatory activities [28,29]. De Roos B et al. have studied the effects of an olive oil production called alperujo extract, in platelet function. As platelets are key players in haemostasis, wound healing, and in inflammatory responses, they concluded that alperujo extract may protect against platelet adhesion and activation and possibly have anti-inflammatory properties [30]. Experimental studies in mice depicted that consumption of olive oil with a natural content of phenolic compounds attenuates adipose tissue hypertrophy and inflammation and exerts anti-atherosclerotic effects [31]. Debbabi M. et. al., showed the positive role of MD focusing their study on the oleic acid (OA), which is a major ingredient of olive and argan oils. Moreover, they extend their studies to the effect of docosahexaenoic acid (DHA) found also in fatty fishes which was able to reduce the 7KC-induced cytotoxicity [32]. Yarla NS et al. in their study on the effect of olive oil (OO) on cytokines indicate that the level of postprandial TNF-α and IL-6 is affected by body mass index (BMI). A long-term olive oil intake has an anti-inflammatory effect through crosstalk between adipose tissue, liver, skeletal muscle, and brain. In this context, the role of olive oil on immune-mediated inflammatory responses involved in obesity and frailty deserves further investigation [33].

### 3.4. Polyphenols and Neutrophil Activity

Polyphenols, such as oleacein (3,4-DHPEA-EDA, 3,4-dihydroxyphenylethanol-elenolic acid dialdehyde), are believed to play a role in the prevention of cardiovascular diseases. As it is known, there is a raise of neutrophil mediators in acute myocardial infarction. The effect of oleacein was determined on neutral endopeptidase (NEP) activity, as well as on other functions of neutrophils in humans. Oleacein concentrations suppress considerable expression of CD11b/CD18 and CD62L on neutrophils surface. NEP activity, elastase release, MMP-9, and IL-8 release were also inhibited. Thus, olive oil seems to have a protective effect against endothelial injuries [34]. Footballers during the acute exercise in training season showed augmentation of the antioxidant defenses of the neutrophils such as catalase, glutathione peroxidase, and glutathione reductase enzyme activities. During the acute training, all oxidative damage markers were decreased in neutrophils [35].

As diet supplementation with omega-3 fatty acids could influence the oxidative equilibrium, enhancing a pro-oxidant status the aim was to determine the effects of diet supplementation with docosahexaenoic acid (DHA), training, and acute exercise on oxidative balance in neutrophils [35]. Diet supplementation with docosahexaenoic acid (DHA) improves, in general, the antioxidative status [36].

### 3.5. Lycopene’s Value

Carotenoids, such as Lycopene are found in vegetables and fruits namely tomato, watermelon, and guava contain increased amounts and dietary consumption of lycopene reduces the oxidative stress [37]. Its anti-inflammatory and protective properties are linked to the large presence of omega-3 polyunsaturated fatty acids, vitamins, but especially to the constituents of extra virgin olive oil: oleic acid, phenolic compounds and olecanthal [38]. It has been shown that the MD could reduce disease activity, pain, and stiffness in patients with inflammatory arthritis and may thus constitute a valuable support for patients suffering from these diseases. As a natural antioxidant, lycopene can alleviate oxidative stress and suppress inflammation and could have the potential to reduce mortality in people with systemic lupus erythematosus (SLE) [39]. Moreover, Buyuklu M et al., in an experimental model of contrast-induced nephropathy (CIN), have demonstrated the favorable effects of Lycopene as an anti-inflammatory, anti-autophagic, and anti-apoptotic agent [40]. Also, Lycopene inhibits IGF-I signal transduction and growth in normal prostate epithelial cells by decreasing DHT-modulated IGF-I production in co-cultured reactive stromal cells [41].

**Figure 1:**
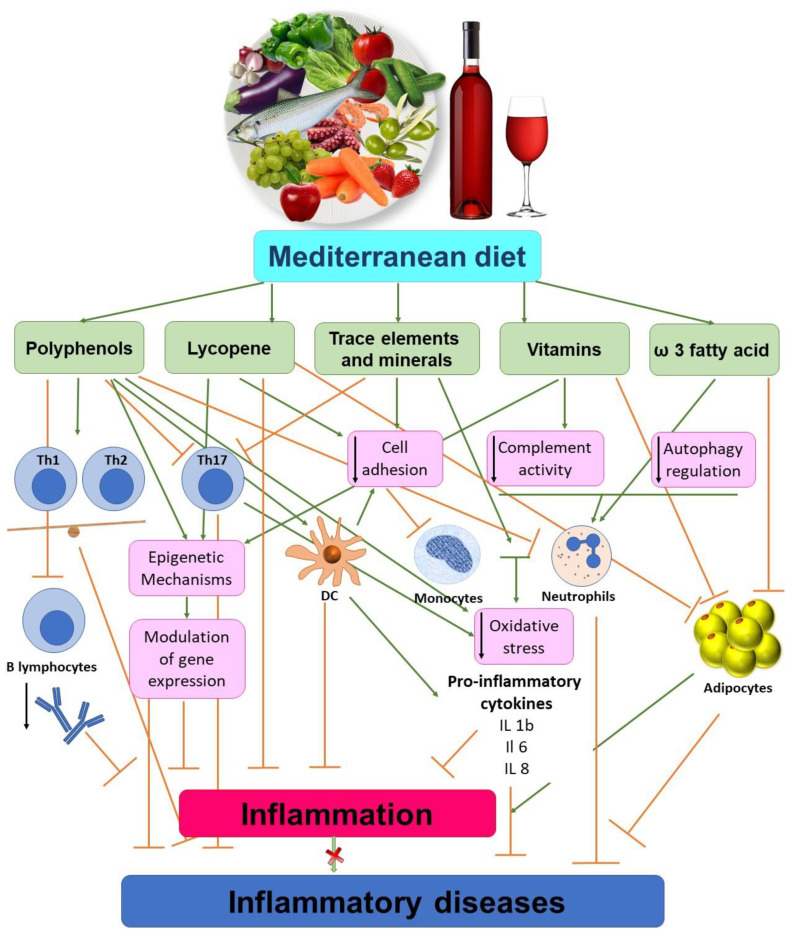
The Mediterranean diet (MD) as anti-inflammatory properties. The MD is rich in antioxidants, trace elements, minerals and vitamins which have anti-inflammatory properties. The MD acts by improving autophagy and Th cells imbalance. Moreover, components of MD down-regulates the expression of cell adhesions molecules like VCAM, ICAM, and E-selectin in circulating immune cells and regulates endothelial dysfunction. On the other hand, the consumption of alcohol increases the risk of serious diseases (liver disorders, pancreatitis, cancer).

Among others, age-related macular degeneration (AMD) is a severe disease leading to blindness in aged people. As known, Lycopene possesses both antioxidative and anti-inflammatory properties and inhibits ICAM-1 expression and NF-κB activation by Nrf2-regulated cell redox state in human retinal pigment epithelial cells [42]. Obesity is characterized by a fibroblast growth factor 21 (FGF21)-resistant status and FGF21 production could be adjusted by some bioactive dietary compounds. Mediterranean tomato-based sofrito sauce seems to improve fibroblast growth factor 21 (FGF21) signaling in visceral white adipose tissue of obese animals [43]. As stated, Lycopene shows a beneficial role in multiple diseases due to antioxidative and anti-inflammatory properties. Its advantageous contribution is also extended to chronic diseases [37]. Lycopene improves cognitive functions in Alzheimer’s disease due to this antioxidative effect as it protects the oxidative damage of mitochondrial enzymes and prevents apoptosis [37]. Chronic obstructive pulmonary disease is characterized by systemic inflammation which is alleviated by dietary lycopene [37]. Moreover, lycopene adjusts osteoporosis by enhancing osteoclast activity and decreasing bone damage [37]. Also, lycopene alleviates neuropathic pain by enhancing the expression of Concexin (CX43) in the dorsal cortex of the spinal cord [37]. Lastly, an effect of Lycopene was also registered in SW480 human colorectal cancer cells as an inhibitor of inflammation [44]. Schematic illustration of the Mediterranean diet (MD) as anti-inflammatory properties is presented in Figure 1.

## 4. Mediterranean Diet’s Impact on Epigenetic Mechanisms

There are several epidemiological and clinical evidence that, nutrition patterns notably MD, are associated with development or progression of major human diseases via epigenetic mechanisms. DNA methylation is one of the most studied epigenetic mechanisms that cells use to control gene expression [45,46].

A dietary intervention with MD low-fat diet was conducted in the framework of the CORDIOPREV project and published two years ago [47]. Concluded that as insulin resistance and chronic inflammation are predisposing factors to type 2 diabetes mellitus (T2DM), through “NOD-like receptor pyrin domain containing-3” (NLRP3) inflammasome component of innate immunity, a metabolic stress sensor, modulated by dietary and genetics factors [47].

The epigenetic effects of different dietary patterns seem to be related to the predisposition and development of major human cancers, such as breast, gastrointestinal, and prostate cancer [48]. Intervention studies, the MeDiet study, and the DiMeSa study focused on evaluating the role of MD in a large panel of both plasma and urine biomarkers. Both studies underpin clinical and biological effects of diet. Moreover, the MD food ingredients seem to slightly enhance the metabolism of estrogens in postmenopausal women and eliminate other produced toxic compounds, reducing the risk of breast cancer as well as withdrawal symptoms of estrogen in menopause women [48].

On the other hand, DiMeSa study underlines the innovative industrialization of products with high health potential and market capacities that could be used to produce Mediterranean reinforced foods [48]. In this vein, the production of monocultivar extra virgin olive oils (EVOs) showed a positive effect on cellular and metabolic processes. The production of highly characterized EVOs is promising for human health and prevention of chronic diseases [48].

Deoxyribonucleic acid (DNA) transcription and messenger ribonucleic acid steadiness could be modulated by the DNA sequences aberrations. As stated already, gene expression could be modulated by other epigenetic mechanisms such as DNA methylation, histone acetylation, chromatin structure aberrations and others. The phytochemical phenolic antioxidants modulate the mammalian cellular epigenome leading to different disease states [49].

Telomeres are DNA structures located at the ends of the linear chromosomes which prevent genome steadiness. Cellular replication results to telomeres shortening leading to the cellular cycle arrest known as senescence [50]. Knowledge collected during the National Health and Nutrition Examination Surveys (NHANES) showed an association between optimal nutrition and longevity [51]. A considerable number of adult individuals (n: 4758) ranging between 20–65 years with no known chronic diseases were enrolled in the study and Mediterranean Diet Scores were related to longer telomeres in women only [51]. Recently, the effect of green tea was evaluated in obese patients. Green tea boosts leukocyte telomere length elongation in obese women. Telomere length and BMI showed an inverse association [52].

Mediterranean diet modulates Leukocyte telomere length (LTL), glucose metabolism, and inflammation pattern in coronary heart disease patients. Telomerase RNA Component (TERC) genetic variants have been associated with LTL which is related to aging-associated diseases. Telomerase RNA Component (TERC) interacts with monounsaturated fatty acids repress inflammation and telomere elongation related to cardiovascular disease [53].

The role of cytokines as mediators to severe diseases was largely stated [54]. Different dietary approaches such as Stop Hypertension (DASH) and the Mediterranean diet (MD) were studied and correlated with circulating hs-CRP and IL-17A levels [55]. Patients adopting the MD presented lower serum IL-17A levels, whereas the DASH diet was related to lower serum hs-CRP levels [55]. The impact of olive oil upon the mediators of chronic low-grade systemic inflammation, TNF-α, and IL-6 was reported [33]. Previous studies showed that IL-6 was associated with the obesity, metabolic syndrome, diabetes cardiovascular diseases, as well as in geriatric anorexia, sarcopenia, cachexia, and frailty [33]. Olive oil seems to modulate these situations.

It is believed that Mediterranean basin’s habitants manage to maintain their competent health status even in old age and this might be associated with the MD and adherence to MD by the elderly may boost dendritic cell (DC) function and consequently a more effective immune response [56].

The effect of MD experiences a decrease in adiponectin response related to the male sex. Shifts in cardiovascular risk factors were also noticed and on the contrary, there was not any impact of MD on leptin in both men and premenopausal women [57]. Furthermore, following considerable weight loss after a hypocaloric diet, the mutant allele (A1359) appears to be associated with a decrease of leptin and IL-6 and resistin in Caucasian people [58].

Different dietary approaches of Mediterranean diets supplanted with extra-virgin olive oil or nuts influence the epigenetic methylation pattern inducing beneficial to health changes in several peripheral white blood cells genes. Those shifts are related to diabetes, metabolism issues, signal transduction, and inflammation [59].

## 5. Chronic Diseases and Mediterranean Diet Interventions

A bulk of scientific information refers to the potential beneficiary effect of MD upon several non-communicable diseases. In Southern Europe, people consuming Mediterranean food products rich in olive oil displayed lower incidence of multiple sclerosis. In an animal model of multiple sclerosis which is the experimental autoimmune encephalomyelitis, the influence of dry olive leaf extract was investigated [60]. Dry olive leaf extract reduced the disease duration and other parameters, such as cumulative disease index and maximal clinical score. Furthermore, diminution was observed in the production of IL-17 and IFN-gamma by showing a beneficial effect in the experimental autoimmune encephalomyelitis model of rats [60].

### 5.1. Autoimmune Diseases

Psoriasis is a skin disease associated with a multifactorial profile, as immunological status, environment, and genetic patterns [61,62,63,64,65,66,67]. Nutrition holds a key role as different aliments interact for the development of psoriasis depicted in several studies implicating gut microbiota as well [66,67,68,69,70,71]. The Mediterranean diet (MD) seems to have a positive healthful impact in the development of psoriasis [72,73]. By using same-gender full siblings as controls for obesity, authors showed the positive correlation of psoriasis severity with BMI in obese patients [74]. Similarly, increase of BMI was related to greater psoriasis risk onset and severity of the disease [75]. In the same light, a conducted multicenter study for obese children showed a correlation with psoriasis as 37.9% of those children developed the disease and also BMI correlated with the severity of psoriasis [76]. The IL-17-producing cells are involved in the pathogenesis of psoriasis [77] and drug pharmacokinetics are influenced by food patterns [78]. As stated previously, different dietary approaches were correlated with circulating hs-CRP and IL-17a levels [55].

Compound of Mediterranean diet and pharmacological treatment decrease the disease activity and improve quality of life in patients with autoimmune disorders such as rheumatoid arthritis and SLE [79,80]. Specifically, beneficial effects of the Mediterranean diet on prevention, treatment, reducing symptoms of pain, and enhancing physical activity in patients with rheumatoid arthritis were observed [79].

### 5.2. Hyperuricaemia

It is widely accepted that diet habits play a role in the development of hyperuricemia as well as in exacerbating of disease [81,82,83]. The risk of attacks is increased after high intakes of purine-rich foods. Urate levels also rise markedly after high intakes of fructose-rich food, red meat, and alcohol. On the other hands, Kontogianni M et al., report that the Mediterranean diet is associated with lower serum UA levels and lower likelihood of hyperuricaemia [82]. In addition, low-fat dairy products, whole grains, nuts, and legumes, as well as fruits and coffee are associated with a decreased risk of gout [84]. Furthermore, studies have shown that vitamin C supplements reduce hyperuricaemia [85,86]. On the contrary, a recent Cochrane systematic review failed to uncover a clinically significant reduction in serum uric acid levels after vitamin C supplementation in comparison with the decrease linked to the use of standard urate-lowering agents [87].

### 5.3. Cardiovascular Disease

Several studies showed the positive effects of the MD on cardiovascular risk factors: blood pressure, body mass index (BMI), blood lipids [88,89]. The components of the Mediterranean diet, such as olive oil being an important ally in cardiovascular disease prevention. Hernáez Á et al., report that the consumption of olive oil reduces the concentrations of low-density lipoproteins (LDL) and increases the concentration of high-density lipoproteins (HDL). Moreover, the MD, improve HDL atheroprotective functions in humans [88]. In addition, olive oil downregulates pro-inflammatory cytokines such as interleukin-6 (IL-6) and tumor necrosis factor (TNF) [90]. Likewise, MD’s better adherence is proven to be correlated to lower risk for stroke as Katherine E. Paterson et al. investigated in observational prospective population-based cohort study of 23,232 men and women [91].

### 5.4. Allergic Diseases and Asthma

Allergic diseases are multifactorial disorders with a wide spectrum of clinical manifestations and symptoms. Genetic predisposition, environmental factors, as well as dietary habits play a critical role in pathophysiology of allergic disorders [6]. The Western diet (WD) that characterized by high fat intake, can cause an increase in airway inflammation. Moreover, WD dietary habits have detrimental consequences, due to the excess consumption of sugar and total energy [6]. Furthermore, a glucose, sucrose, and fructose as components of WD are critically for cell activation and allergic immune response [92]. Contrary to WD, there is clinical and epidemiological evidence that Mediterranean-style diet is associated with lower asthma prevalence in children [93,94]. Moreover, MD intake, which is abundant in the lycopene, reduced airway inflammation in asthmatic patients. Wood, L.G et al., report that just seven days of MD supplementation is able to reduce neutrophil influx and sputum neutrophil elastase activity [95]. Moreover, gut and upper airway microbiota, play a key role in pathogenesis of asthma. The cornerstone of this opinion is the ‘hygiene hypothesis’ which introduced by Strachan in 1989. He postulates that microbial exposure in infancy and childhood is protective against atopy and asthma. [96]. Studies have shown that microorganisms play a protective role in allergic disorders [97,98]. Finally, the fact that MD is characterized by richness of beneficial nutrients, may be the main reason for the inverse association with chronic obstructive pulmonary disease (COPD) according to A.Fischer et al. [99].

### 5.5. Inflammatory Bowel Diseases

Inflammatory bowel diseases (IBD) is a term that include two conditions (Crohn’s disease and Ulcerative Colitis), is characterized by chronic inflammation. In the last decades, the incidence of IBD is increasing worldwide. The pathogenesis of IBD includes genetic predispositions, gut microbiota dysregulation (dysbiosis), and direct influence by environmental triggers [100]. Among the environmental factors, diet plays a cornerstone role by modulating the gut microbiome and influencing epigenetic changes [101]. Therefore, diet could be applied as a therapeutic tool to improve the disease manifestation [102].

## 6. Acute Inflammation-Sepsis

It is well known that patients with chronic inflammatory diseases such as obesity, type 2 diabetes mellitus, et cetera are in high risk for sepsis. In addition, nutrition may play an important role in influencing sepsis risk. The association between the MD and sepsis is unknown. Since MD is associated with high risk of CVD, such as a stroke and prior stroke is closely associated with risk of sepsis, Gray et al. in a recent study entitled: “REasons for Geographic and Racial Differences in Stroke (REGARDS) study” report that moderate and high adherence to MD was associated with a 9% lower risk of severe sepsis [103]. On the other hand, studies have shown that WD is associated with high risk of sepsis. WD intake leads to inflammation and endothelial cell activation [104,105]. Animal model studies have shown that high-fat diets that mimic WD-impaired immune function, escalate inflammation and organ damage after induction of sepsis [106,107].

## 7. Conclusions

It is generally accepted at the moment, that the non-communicable diseases (NCD) namely cardiovascular disease, cancer, depression, diabetes etc. represent the protagonist of the death toll in the whole world. Epidemiological studies exist with regards to an actual link between MD regime and lower incidence of NCD and mortality. Apart from well-established data for CVD, for other non-communicable ones, the beneficial impact is still vague and unclear. Mediterranean diet and its components placed in the family and friends’ landscape, representing a holistic perspective has been under the microscope for the last 50 years. A great number of experimental and clinical studies promote the notion of its eminent role for maintaining health quality with a global impact. Having considered all the above, much needs to be studied and investigated to prove causality, gut microbiome shifts, environmental factors, and potential dietary interventions towards personalized medicine with the adoption of a Mediterranean diet and promote global health.

## Abbreviations

MD	Mediterranean diet
WD	Western diet
CVD	Cardiovascular Diseases
T2DM	Type 2 Diabetes Mellitus
